# Understanding the influence of substrate when growing tumorspheres

**DOI:** 10.1186/s12885-021-07918-1

**Published:** 2021-03-15

**Authors:** Lucía Benítez, Lucas Barberis, Luciano Vellón, Carlos A. Condat

**Affiliations:** 1 Instituto de Física Enrique Gaviola, CONICET, and Facultad de Matemática, Astronomía, Física y Computación, Universidad Nacional de Córdoba, Córdoba, X5000 HUA Argentina; 2 Instituto de Biología y Medicina Experimental, CONICET., Buenos Aires, C1428 ADN Argentina

**Keywords:** Tumor growth, Cancer stem cell, Niche, Substrate, Tumorsphere, Mathematical modelling

## Abstract

**Background:**

Cancer stem cells are important for the development of many solid tumors. These cells receive promoting and inhibitory signals that depend on the nature of their environment (their niche) and determine cell dynamics. Mechanical stresses are crucial to the initiation and interpretation of these signals.

**Methods:**

A two-population mathematical model of tumorsphere growth is used to interpret the results of a series of experiments recently carried out in Tianjin, China, and extract information about the intraspecific and interspecific interactions between cancer stem cell and differentiated cancer cell populations.

**Results:**

The model allows us to reconstruct the time evolution of the cancer stem cell fraction, which was not directly measured. We find that, in the presence of stem cell growth factors, the interspecific cooperation between cancer stem cells and differentiated cancer cells induces a positive feedback loop that determines growth, independently of substrate hardness. In a frustrated attempt to reconstitute the stem cell niche, the number of cancer stem cells increases continuously with a reproduction rate that is enhanced by a hard substrate. For growth on soft agar, intraspecific interactions are always inhibitory, but on hard agar the interactions between stem cells are collaborative while those between differentiated cells are strongly inhibitory. Evidence also suggests that a hard substrate brings about a large fraction of asymmetric stem cell divisions. In the absence of stem cell growth factors, the barrier to differentiation is broken and overall growth is faster, even if the stem cell number is conserved.

**Conclusions:**

Our interpretation of the experimental results validates the centrality of the concept of stem cell niche when tumor growth is fueled by cancer stem cells. Niche memory is found to be responsible for the characteristic population dynamics observed in tumorspheres. The model also shows why substratum stiffness has a deep influence on the behavior of cancer stem cells, stiffer substrates leading to a larger proportion of asymmetric doublings. A specific condition for the growth of the cancer stem cell number is also obtained

**Supplementary Information:**

The online version contains supplementary material available at (10.1186/s12885-021-07918-1).

## Background

For some time, it has been known that the presence of cancer stem cells (CSCs) is important for the development of many solid tumors [1–6]. According to the CSC hypothesis these cells are often crucial for the development of resistance to therapeutic interventions [7, 8]. In healthy tissues the proportion of stem cells is small; homeostatic equilibrium is maintained through the signals that the stem cells receive from their niches. The onset of cancer is likely to destroy this equilibrium and cancerous tissues may exhibit a higher proportion of stem cells than normal tissues [9]. This increased proportion of cancer stem cells may underlie the aggressive behavior of high-grade tumors [2, 10]. As recently explained by Taniguchi et al. [11], the cross-talk between tumor initiating (stem) cells and their niche microenvironment is a possible therapeutic target. Understanding the nature of the interactions between CSCs and their environment is therefore important for the development of effective intervention procedures.

Interesting mathematical models have been developed to explain various properties of stem-cell-driven tissue growth. Stiehl and Marciniak-Czochra proposed a mathematical model of cancer stem cell dynamics to describe the time evolution of a leukemic cell line competing with healthy hematopoiesis [12]. This group later provided evidence that the influence of leukemic stem cells on the course of the disease is stronger than that of non-stem leukemic cells [13]. Yang, Plikus and Komarova used stochastic modeling to explore the relative importance of symmetric and asymmetric stem cell divisions, showing that tight homeostatic control is not necessarily associated with purely asymmetric divisions and that symmetric divisions can help to stabilize mouse paw epidermis lineage [14]. Recently, Bessonov and coworkers developed a model that allowed them to determine the influence of the population dynamics on the time-varying probabilities of different cell fates and the ascertainment of the cell-cell communication factors influencing these probabilities [15]. These authors suggest that a coordinated dynamical change of the cell behavior parameters occurs in response to a biochemical signal, which they describe as an underlying field. Here we will describe the effects of cellular interactions using nonlinear terms instead.

Live cells are generally sensitive to substratum rigidity and texture [16]. A growing tumor must compete for space with the surrounding environment; the resulting mechanical stresses generate signals that impact on the tumor cells. Cells integrate these mechanical cues and respond in ways that are related to their phenotype. Their active response may also lead to phenotype modifications [17, 18]; in fact, mechanical cues generated by the environment can trigger cancer cell invasion [19]. Environmental stiffness may then be associated with tumor progression, a process that can also be promoted by mechanically activated ion channels [20].

What is the influence of the mechanical environment on cancer stem cells? At each generation, CSCs divide symmetrically, generating either two new CSCs or two differentiated cancer cells (DCCs), or asymmetrically, generating one CSC and one differentiated cancer cell [7, 21]. Quorum sensing controls differentiation of healthy stem cells, but it is thought to be altered in cancer stem cells [22]. Mechanical inputs are an important component of the altered control mechanism and can be assumed to play a role in the fate of the cancer stem cells. In vitro experiments have been designed to probe the influence of mechanical stresses of various types on tumor cells. The solid-stress inhibition of multicellular spheroid growth was already demonstrated by Helmlinger and coworkers in 1997 [23]. The results of these experiments were shown to follow allometric laws [24]. Interestingly, Koike et al. showed that spheroid formation with Dunning R3327 rat prostate carcinoma AT3.1 cells is facilitated by solid stress [25].

A study by Cheng et al. suggested how tumors grow in confined locations where levels of stress are high, showing that growth-induced solid stress can affect cell phenotype [26]. Using spheroid cell aggregates, Montel et al. showed that applied pressure may be used to modulate tumor growth [27] and observed that cells are blocked by compressive stresses at the G1 checkpoint [28]. The organization of cells in a spheroid is modified by physical confinement [29], which likewise modifies the proliferation gradient [30]. The stiffness of hydrogels has been shown to determine the shape of tumor cells, with high stiffnesses leading to spheroidal cells, a feature known to occur in in vivo tumors [31]. By studying the behavior of adult neural stem cells under various mechanical cues, Saha et al. showed that soft gels favored differentiation into neurons while harder gels promoted glial cultures. Importantly, they also showed that low substrate stiffness inhibited cell spreading, self-renewal, and differentiation [32]. Osteocyte-like cells were shown to significantly induce compaction of tumor spheroids formed using breast cancer cells [33]. Matrix stiffness was shown to affect, through mechanotransduction events, the osteogenic outcome of human mesenchymal stem cell differentiation [34]. HeLa cells were used to show that both an attractive contact force and a substrate-controlled remote force contribute to the formation of large-scale multicellular structures in cancer [35].

Fifteen years ago, Discher, Janmey, and Wang not only explained that the stiffness of the anchoring substrate can have a strong influence on the cell state, but they also indicated that stem cell differentiation may be influenced by the nature of the substrate [36]. It is relevant that naive mesenchymal stem cells were shown to commit to various phenotypes with high sensitivity to tissue elasticity: They differentiate preferably into neurons and osteocytes if they are cultured on soft and rigid matrices, respectively [37]. On the other hand, human mesenchymal stem cells adhere onto precalcified bones, which are softer than calcified bones [16]. It is also known that hydrodynamic shear stress promotes the conversion of primary patient epithelial tumor cells into specific cancer stem-like cells [38]. Smith et al. found that the mechanical context of the differentiation niche can drive endothelial cell identity from human-induced pluripotent stem cells, showing that stiffness drives mesodermal differentiation, leading to endothelial commitment [39]. Thus, microenvironments help specify stem cell lineages, although it may be difficult to decouple the influence of mechanical interactions and surface topography and stiffness from biochemical effects [16, 39]. Since they are grown in the absence of the complex signaling system prevalent in the environment of real tumors, tumorspheres, spheroids formed by clonal proliferation out of permanent cell lines, tumor tissue, or blood [40], are suitable candidates to probe the influence of mechanical stimuli on stem-cell-fueled cancer growth.

Wang et al. cultured breast CSCs on soft and hard agar matrix surfaces, investigating the effects that substrate stiffness has on cell state and proliferation [41]. These authors showed that breast cancer stem cells can be kept in states of differentiation, proliferation or quiescence depending on a combination of adherent growth and stem cells growth factors, but they focused on the experimental possibilities and did not draw conclusions about how these agencies may modify the stem cell niche to lead to the observed behavior. Recently, we developed a two-population tumorsphere model to identify the role of the intraspecific and interspecific interactions that determine tumorsphere growth [18]. Application of our model to three breast cancer cell lines studied by Chen and coworkers [9] indicates that while intraspecific interactions are inhibitory, interspecific interactions promote growth. This feature of interspecific interactions was interpreted in terms of the stimulation by CSCs of the growth of DCCs in order to consolidate their niches and of the plasticity of the DCCs to dedifferentiate into CSCs [18]. Here we use this model to analyze the experimental results of Wang et al. [41], discussing how substrate stiffness influences growth and finding that the concept of cancer stem cell niche is central for its understanding. In the next section we review the model of Ref. [18] and in the following sections we apply it to the results of Ref.[41] and discuss their implications.

## Methods

**Fig. 1:**
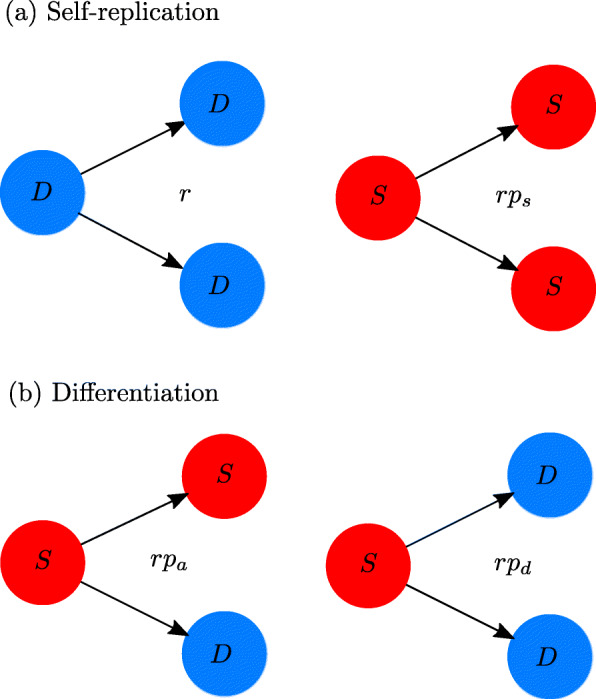
Schematic representation of the cell division outcomes and the intrinsic growth rates. Each of these is given by the product of the basal growth rate *r* and the probability of the respective outcome. **a** Parent cells replicate themselves originating daughter cells in their same class. **b** CSC differentiation occurs in two ways: if asymmetric, the *S* population remains unchanged; if symmetric, *S* decreases
- The members of each subpopulation interact with each other (intraspecific interactions) and with the members of the other subpopulation (interspecific interactions), c.f. Fig 2. These interactions are described by proportionality factors *α*_*ij*_ whose signs and magnitudes quantify the number of cells that are created in the system due to interactions with preexisting cells. The indices *i* and *j* may represent *S* or *D*. They indicate either intraspecific (*i*=*j*) or interspecific (*i*≠*j*) interactions.

We model mathematically the growth of a tumorsphere considering two cell populations: Cancer stem cells (*S*) and differentiated cancer cells (*D*). By including in the last class all cells with any degree of differentiation we can isolate the role played by the stem cells. We further assume that:

- The single basal growth rate *r* characterizes the timescale of the system. By construction, it matches a priori the population doubling time (PDT) of the DCCs. This provides a more suitable description than the previous model with two basal growth rates [18] since, in general, it is not possible to discriminate between these rates in experiments such as that of Ref. [41].
- When a CSC undergoes mitosis there is a probability *p*_*s*_ that two new CSCs are generated, a probability *p*_*d*_ that two DCCs are generated, and a probability *p*_*a*_ that there is an asymmetric division. Because of normalization, *p*_*a*_=1−*p*_*d*_−*p*_*s*_. These probabilities should be multiplied by the basal growth rate *r*, see Fig. 1, in such a way that it is possible to reasonably model the effective creation rates of new cells.

**Fig. 2:**
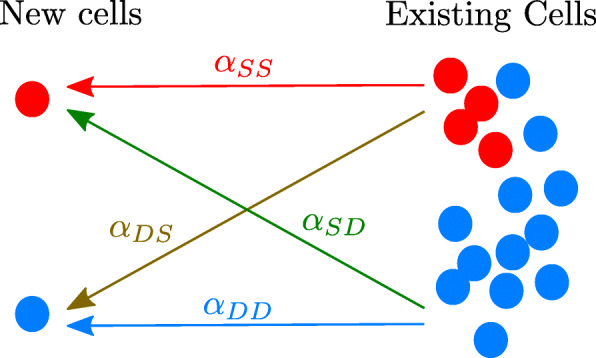
The signs of the coefficients *α*_*ij*_ indicate whether the interactions promote or inhibit growth. Cells already present in the tumorsphere either favor (*α*_*ij*_<0) or hinder (*α*_*ij*_>0) the production of new cells. Arrows indicate the influence of each population on the newborn cells

We can describe the evolution of the two interacting populations by generalizing the standard equations for two competing species (see, p. ej. [42], p. 67).

1a $$ \frac{dS}{dt}=r[ p_{s} S]\left\lbrace \frac{p_{s}- p_{d}}{ p_{s}} - \alpha_{SS}S - \alpha_{SD}D \right\rbrace,  $$

1b $$ \frac{dD}{dt}=r [D+ (1+p_{d}- p_{s})S]\left\lbrace 1-\alpha_{DD}D - \alpha_{DS}S \right\rbrace.  $$

The first term inside the braces on the right-hand side of Eq. (1a) corresponds to the net intrinsic creation of new CSCs (symmetric CSC divisions minus divisions yielding two DCCs). Note that asymmetric divisions do not change the number of cancer stem cells, but symmetric differentiation removes the parent CSC from the *S* population, as illustrated in Fig. 1. The second and third terms correspond, respectively, to the effects on the CSC population of the interactions with other CSCs and with differentiated cancer cells.

The factor in the square brackets on the right-hand side of Eq. (1b) is proportional to the rate of creation, in the absence of interactions, of differentiated cells due to the division of other DCCs (first term), plus the asymmetric division and differentiation of CSCs (second term). The first term between braces corresponds to the interaction-free growth of the system. The second and third terms represent, respectively, the influences of the other DCCs and of the CSCs on the differentiated cancer cell population. The effect of cell-cell interactions on cell creation is assumed to be proportional to the abundances of the populations emitting the signals and of those receiving them; therefore, the corresponding terms are quadratic in the populations. The interaction strengths are represented by the coefficients *α*_*ij*_. Negative interaction coefficients (*α*_*ij*_<0) describe growth-promoting interactions, e.g. the *j* population promotes the growth of the *i* population. Positive values of *α*_*ij*_ describe the growth inhibition of population *i* by population *j*. In particular, as shown in Fig. 2, *α*_*SS*_ tells us how CSCs promote/inhibit the creation of new CSCs, *α*_*DD*_ tells us how DCCs promote/inhibit the creation of new DCCs, while *α*_*DS*_ informs us about the influence of CSCs on the generation of new differentiated cancer cells and *α*_*SD*_ the influence of DCCs on the generation of new cancer stem cells.

There are no analytic solutions for these differential equations. Their numerical solutions yield the time evolution of both subpopulations, *S*(*t*) and *D*(*t*). In Additional file 1 we summarize some properties of Eqs. (1) and their solutions that we will use in our analysis.

### Fitting with the model

The data sets correspond to the total cell number *T* in the spheroids. Thus, we fit the data with *T*=*S*+*D*, where *S* and *D* are the numerical solutions of the system of Eqs. (1). Thereby, our model allows us to obtain information on the dynamics of the CSC and DCC subpopulations and, in particular, on the time evolution of the CSC fraction, from data corresponding to the whole spheroid. Due to the scarcity of data points and the ensuing difficulties of the optimization problem, fitting our model to the data leads to different sets of possible parameter values. To obtain the optimal set, we use a random grid search (RGS) algorithm. The RGS algorithm consists in randomly sweeping some domain for initial conditions in parameter space. In our case, such a domain is bounded by physically reasonable assumptions such as that the values of probability be *p*_*i*_∈[0,1] with *i*≡*s*,*d* and the growth rate *r*>0. Also we ask for the outcome of the fitting process to give normalized positive probabilities, positive populations in the range of validity of the data, and fractions of the order of the ones reported by Wang et al. We then collect in a histogram all the parameters that have a relative error lower than 5% when fitting the data points. To do this we define a relative error measure given by the nonlinear estimator

$$R=\frac{1}{n}\sum_{i=1}^{n} \left(\frac{y_{i}-Y(t_{i})}{y_{i}} \right)^{2}. $$

Here *n* is the number of data points, *y*_*i*_ the data value at time *t*_*i*_, and *Y*(*t*_*i*_) the function value obtained by fitting the data. This estimator is the same as the function we minimize through the fitting process (the classical *R*^2^ parameter also used as a minimization - objective function, is not a good reporter for a nonlinear problem). A first selection criterion of the RGS algorithm ensures that no accepted parameter set has an accuracy below 95%. A consistent statistical interpretation of the process requires that the order of magnitude and, especially, the sign of each parameter be the same in all realizations. Therefore, even if different combinations of the fitting parameters yield acceptable descriptions of the experimental results, the qualitative mechanisms that control spheroid growth can be satisfactorily identified. We thus find a distribution for each parameter and select the median as its representative value.

## Results

### The initial stages

First, let us answer the following question: Given that we start tumorsphere growth from a small CSC seed, what is the minimum size *S*_*m*_ needed for this seed to guarantee CSC population growth? By setting *D*=0 in Eq. (1b), we see that there are two cases:

- If differentiation is inhibited, *p*_*s*_>*p*_*d*_, as in the case of the *soft* and *hard* experiments discussed below, the linear term dominates and the initial seed may be arbitrarily small: a single cancer stem cell may generate a tumorsphere.
- If *p*_*s*_<*p*_*d*_, it is easy to see that the condition for initial CSC number is that the quadratic term be large enough, i.e. *S*_0_>*S*_*m*_, with
  2 $$ S_{m} = \frac{p_{s} - p_{d}}{ \alpha_{SS} p_{s}}.  $$

We thus need *α*_*SS*_<0 : The CSCs must cooperate to yield additional cancer stem cells starting from a pure CSC seed. A larger cooperative interaction implies that we can use a smaller seed. In this case, the intraspecific interaction coefficient *α*_*SS*_ plays a key role in the growth determination from the very beginning of the process. It is worth mentioning that in the experiments discussed here the conditions *p*_*s*_<*p*_*d*_ and *S*_0_>*S*_*m*_ are never satisfied simultaneously. Usually, *p*_*s*_ is smaller than *p*_*d*_, and there is a minimum number of stem cells required to ensure stem cell growth. But if a differentiation - inhibiting agent is added to the system, increasing *p*_*s*_, a single cancer stem cell may suffice to generate growth. As shown in Additional file 1, we can linearize our equations to describe the initial evolution of a small system, finding that the trajectory in the *S*−*D* plane starts as,

3 $$ D(t) = \frac{(D_{0} + S_{0})}{S_{0}}S(t)^{1/(p_{s} -p_{d})}- S(t),  $$

where *S*(0)=*S*_0_ and *D*(0)=*D*_0_. Initially, if *p*_*d*_>*p*_*s*_, the number of differentiated cells increases, while the number of stem cells decreases, and the representative point gets close to the *D*-axis. If there is growth in the stem cell subpopulation, it is due to the nonlinear terms.

Our model therefore generates a simple analytical description of the early stages of tumorsphere evolution and specifies the conditions for a successful implantation of the initial cancer stem cell seed. Unless a potent anti-differentiation agent is added to the growth medium, we expect the differentiation probability *p*_*d*_ to be larger than *p*_*s*_. If so, our Eq. (2) predicts the minimum number of stem cells needed to initiate successful spheroid growth. This number depends only on *p*_*s*_, *p*_*d*_, and the intraspecific interaction between cancer stem cells, which must be cooperative. Weak cooperation or a small *p*_*s*_ would mean that the tumorsphere must be started from a large nucleus.

In the next section we review the experimental results reported in [41] and determine the model parameters.

### Experimental data

We used our model to analyze the results of Wang and coworkers [41]. These authors studied the growth of breast cancer cell cultures belonging to three different cell lines: MCF7, MDA-MB-231 and MDA-MB-435. For each of these tumor lines they grew tumorspheres using three different environmental conditions, as detailed below. In all cases the spheroids initially have 4-5 cells that originate from a single CSC. Since only the MDA-MB-231 cell line yielded *bona fide* round spheroids for all three experimental specifications, we will use this line to compare our findings with the experimental results. To facilitate the implementation of the model presented in [18], we report the data in terms of cell numbers.

### Soft substrate

**Fig. 3:**
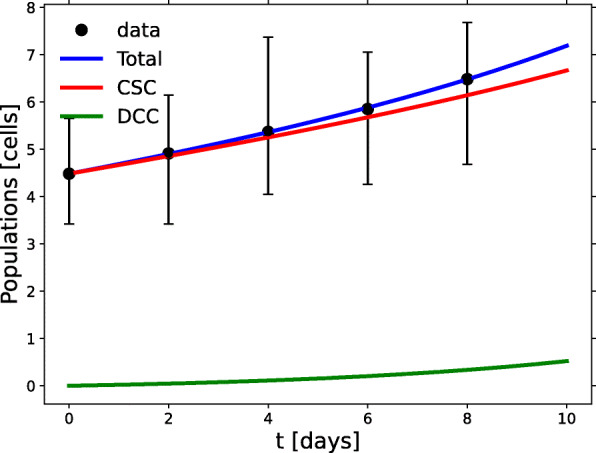
Growth in the *soft* experiment. Data-based model results for the CSC (red) and DCC (green) spheroid subpopulations grown on a soft substrate. The blue line fits the total cell population

In the *soft* experiment, cells were cultured using soft (0.05%) agar as the matrix surface for cell contact. Differentiation inhibitors were added to the growth medium to increase the CSC fraction. Under these conditions there is little incentive for the stem cells to either duplicate or leave their quiescent status. Only the tendency to build a suitable niche may break the quiescence. Hence their small basal growth rate. As a result, a slow exponential growth of CSCs prevails in the early stages of tumorsphere growth as depicted in Fig. 3. Such behavior can be predicted as shown in Eq. 3 and Eqs. (A3) and (A4) in Additional file 1, but the basal growth rate is so small that the process appears to be almost linear. The CSCs population (red line) is always much larger than its DCC counterpart; as a matter of fact, Wang et al. reported a 95% of CSC at day 8 with a low growth rate. The distribution of the fitting values generated by the RGS method is shown in Additional file 2, Fig. A. Note there, and in Table 1, the very high (close to unity) value of *p*_*s*_, the positive sign of the intraspecific interaction coefficients and the negative sign of the interspecific interaction coefficients.

**Table 1:** The three chosen parameter sets obtained from fits to the experimental data

Constant	Units	Soft	Hard	Control
*r*	[1/days]	0.0685	0.1335	0.0240
*p*_*s*_	none	0.9701	0.7124	0.1646
*p*_*d*_	none	0.0019	0.0000	0.3911
*α*_*SS*_	[1/cells]	0.0873	-0.0456	0.0028
*α*_*SD*_	[1/cells]	-0.4185	-0.5280	0.0266
*α*_*DS*_	[1/cells]	-0.2061	-0.1376	-1.0683
*α*_*DD*_	[1/cells]	0.3668	1.8329	-0.3087
$\frac {S}{S+D}|_{8days}$	none	0.9479	0.913	0.141

### Hard substrate

**Fig. 4:**
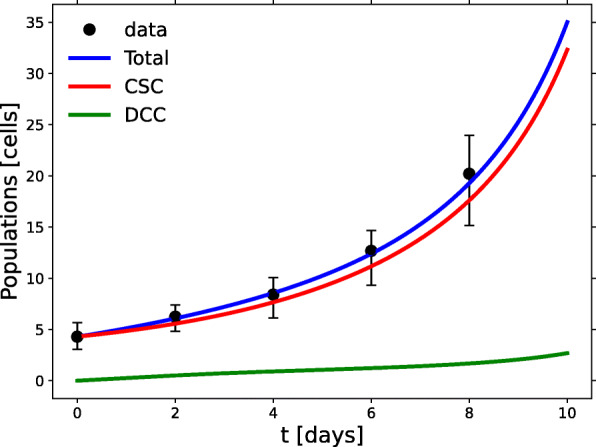
Growth in the *hard* experiment. Data-based model results for the CSC (red) and DCC (green) spheroid subpopulations grown on a hard substrate. The hard substrate yields a faster growth rate than the soft substrate, and, at late times, a higher fraction of CSCs

In the *hard* experiment, cells were cultured using hard (30%) agar as the contact matrix surface. Differentiation inhibitors were also added to the growth medium. For this experiment we expect the model to describe a high fraction of CSCs, as in *soft*, but now with a higher proliferation rate. Applying the RGS method to this data set, we see that this is indeed so (see Table 1 and Additional file 2, Fig. B for the resulting parameters), obtaining the curves depicted in Fig. 4. At early times, growth is nearly linear, as observed in *soft*, but only for the first four days, speeding up afterwards. The CSCs outnumber the DCCs, reaching 91% of the cell population by day 8, consistently with the results reported in [41]. This fraction is a little lower than in *soft* but would become much larger than that at later times.

The symmetric CSC reproduction probability is still high, but noticeably lower than in *soft*, and the basal rate is twice that in *soft*. The interspecific interaction coefficients are negative, as in *soft*, but the CSC intraspecific interaction coefficient is now negative, too.

### Control substrate

**Fig. 5:**
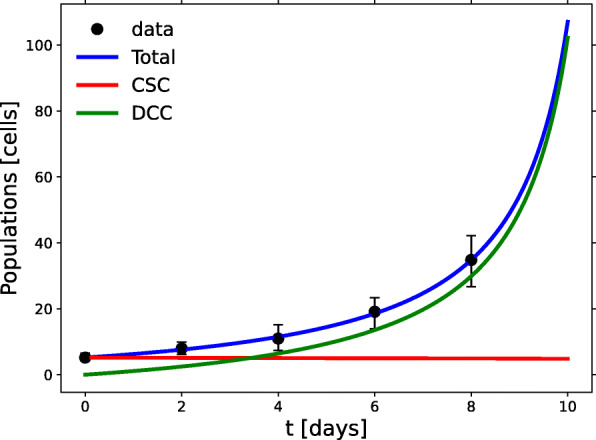
Growth in the *control* experiment. Fitting the *control* medium data predicts unlimited growth, faster than in either *soft* or *hard*, but now driven by the DCCs. The number of CSCs does not increase

In the *control* experiment, cells were cultured using hard (30%) agar as the contact matrix surface, but no differentiation inhibitor was added to the medium. The stem-cell promoting factors EGF and bFGF were replaced by neutral serum and the cells were grown on a *hard* substrate [41]. In this case, although the spheroid cannot preserve its spherical shape at late times, a fitting attempt, shown in Fig. 5, is informative (the corresponding boxplots are shown in Additional file 2, Fig. C).Although the CSC number remains nearly constant, the DCCs can proliferate indefinitely, leading to fast overall growth.

All the new relevant information obtained from fitting the experimental data is summarized in Table 1, where we report the values of the parameters of our model. Those values have an accuracy of 98% and their correspondig distributions are reportedd in Additional file 2. Furthermore, in Table 2, we report some quantities, derived from parameters in Table 1 that will be useful in the following sections.

**Table 2:** Useful derived quantities from some values of Table 1

Constant	Units	Soft	Hard	Control
1/*r*	[days]	14.59	7.49	41.66
*rp*	[1/days]	0.0663	0.0951	-0.0054
$\frac {1}{rp}$	[days]	15.08	10.52	-185
*p*=(*p*_*s*_−*p*_*d*_)	none	0.9682	0.7124	-0.2265

## Discussion

In normal tissues, homeostasis is guaranteed by factors secreted by differentiated cells that inhibit the division and self-renewal of stem cells [22, 43]. Cancer stem cells may partially escape these controls, but their activity is still influenced by their environment. In non-anchored cells, as is the case analyzed in the present work, clustering of most integrins on the plasma membrane by ECM molecules, and thus FAs formation, is lost. In normal cells, such events are sufficient to trigger anoikis, but upregulation of specific integrins can confer anoikis resistance. For instance, *ανβ*3 integrin has the ability to maintain receptor clustering in non-adherent cells (reviewed in Hamidi and Ivaska [44]). Interestingly, the MDA-MB-231 cells used by Wang et al. [41] are an *ανβ*3 integrin-overexpressing breast cancer cell line and highly dependent on *ανβ*3-emanating signals for proliferation and survival [45, 46], and it is likely that changes in the stiffness of the substratum may alter integrins clustering and consequently, cell proliferation.

We would like to emphasize some experimental facts from Ref. [41] that are useful for the interpretation of the results:

- A remarkably high percentage (>95%) of the cells cultured under *soft* and *hard* conditions with growth factors express the stem cell marker Oct4, which is frequently used as a marker for undifferentiated cells. Oct-4 expression must be tightly regulated; too much or too little leads to cell differentiation.
- The *soft* and *control* experiments show low activity of telomerase, a marker for proliferation. The higher telomerase activity exhibited by *hard* indicates a faster growth rate. This is consistent with the expression rates of Ki67-positive, which are close to 90% for *hard* and minimal in the other cases.
- The high (95%) CSC fraction and low (<5%) proliferation rate observed in *soft* at day 8 suggest a population largely consisting of quiescent CSCs. The proliferative fraction was higher in *hard*.
- In *control*, markers indicate a strong dominance of the differentiated state. The stem cell fraction (∼5%) and proliferation rate (5% according to KI-67 and 22% according to flow cytometry) are both low.

In the *soft* and *hard* experiments, cells must adapt to the restrictions imposed by the application of the stem cell maintenance factors EGF and bFGF. We especially extracted information about the cell dynamics from four features: the basal growth rate, the CSC fraction, and the intraspecific and interspecific interaction parameters. The parameter sets resulting from fitting the model to the *hard*, *soft* and *control* experiments, summarized in Table 1, are quite different. We next separately interpret the results of each experiment.

### Soft substrate

The computed basal growth rate *r* is 0.069 day ^−1^, which means that the PDT is close to 15 days. This is consistent with the results obtained by Wang et al. [41] using flow cytometry, but somewhat longer than typical cancer stem cells doubling times, which range from 3 to 11 days, depending on tumor type and culture conditions [47, 48]. DCCs normally reproduce faster but, because in this model *r* represents the average growth rate of the whole population, we recover a PDT consistent with that of the dominating CSCs. This lends support to our modeling assumption of a single basal growth rate.

Quiescence is the prevalent state of the stem cells. Since their function is to replenish dead or damaged cells, they enter the cycle when their niches signal the need for new cells. In *soft* (and in *hard*) the addition of differentiation inhibitors implies that the CSCs always record low DCC populations. This drives them into the cycle, where they divide but, prevented from differentiating, overwhelmingly generate new CSCs. Differentiation is very unlikely (*p*_*d*_=0.0019) and we may neglect it to simplify the analysis. If we do this, there is no linear contribution of the CSCs to DCC generation. With the parameter values in Table 1, the equilibrium point where the two kind of cells coexist is located at (*S*^∗^,*D*^∗^)=(−21.7,−7.0) cells. This point lies in the third quadrant indicating that there is no physical/biological coexistence of the two populations. There are no attractors in the first quadrant and all trajectories diverge. This confirms that CSCs lose their normal quiescent state in a continuous (and futile) attempt to produce more DCCs.

The (positive) intraspecific interaction coefficients *α*_*ii*_ are here directly related to the individual maximum population sizes of the respective subpopulations. If we assumed that the two subpopulations did not interact, *ij*=0, *i*≠*j*, Eq. (1a) would read:

4 $$ \frac{dS}{dt}=rS\left[(p_{s} - p_{d})- p_{s} \alpha_{SS} S \right],  $$

which is a logistic equation that leads to a maximum population size *S*_*c*_=(*p*_*s*_−*p*_*d*_)/(*p*_*s*_*α*_*SS*_)≃12 cells. In this way, from Eq. (1b), we obtain *D*_*c*_=1/*α*_*DD*_≃2 cells for the DCCs, which is six times smaller than *S*_*c*_. Therefore, if there were no interactions between subpopulations our model would predict a 14-cell spheroid, a size that would be reached by day 17. Interactions between the populations are needed to understand the faster growth observed in the experiment. The negative values of the interspecific interaction coefficients, *α*_*ij*_<0, *i*≠*j* lead to a positive feedback loop: An increase in one subpopulation drives an increase in the other. The numbers in Table 1, especially the relatively large value of *α*_*SD*_ (5 times that of *α*_*SS*_), and the relatively low value of *α*_*DS*_ (less than half of *α*_*DD*_), indicate that this interplay favors a net increase in CSC number but is not strong enough to lead to an increase in DCC number.

**Fig. 6:**
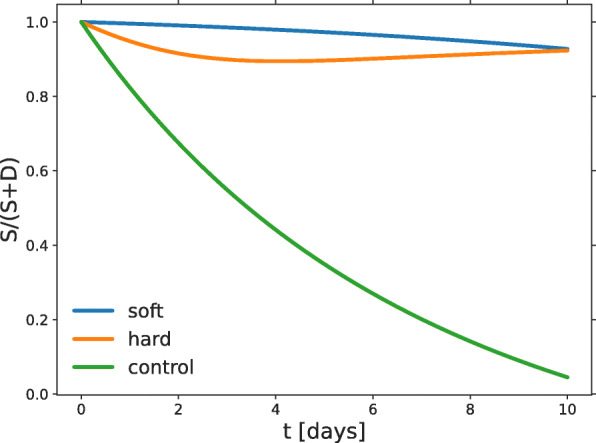
CSC fractions. Time evolution of the cancer stem cell fraction predicted by the model for the three experiments. In both *soft* and *hard* the stem cell fraction remains very high, since the stem cell maintenance factors are a barrier to DCC generation. In *control* the differentiation barrier is not present and the stem cell fraction decreases towards the value corresponding to the niche

The feedback loop mechanism is activated to generate a suitable niche, which requires a low *S*/(*S*+*D*) fraction. The inhibition of differentiation causes the CSCs to continuously reproduce in a frustrated attempt to recreate the DCC population required by the niche. Since the population equilibrium corresponding to a stable niche is never reached, cycling CSCs seldom return to quiescence. In Fig. 6, the fraction *S*/(*S*+*D*) is depicted for the three experiments up to day ten. Due to the inhibitor’s efficacy, this fraction falls very slowly for the *soft* and *hard* substrates (light blue and orange lines, respectively), but decays freely in the *control* environment.

### Hard substrate

When the substrate hardens the environmental conditions that mediate cell-to-cell signaling change and the CSC phenotype becomes more amenable to proliferation, as seen by comparing Figs. 3 and 4. The growth rate *r*, which still represents the PDT of the CSCs because of their prevalence, is twice as large as that corresponding to growth on the *soft* substrate. Our interpretation is that increasing the substrate hardness alters the CSC phenotype required to reach the cell fraction that regulates niche size. As in *soft*, the CSCs try to increase the DCC population but now they are immersed in a different environment. The duplication of the growth rate *r*, the reduction of the symmetric duplication probability *p*_*s*_, and the emergence of a large fraction (>50%) of asymmetric divisions indicates that the direct effect of the differentiation-inhibiting factors is weaker than in *soft*. Indirect effects appear through the interspecific coefficients, especially the relatively large and negative (-0.53) *α*_*SD*_. As Fig. 6 shows, by day 8 the DCC fraction is not much larger than in *soft*, indicating that the attempt to establish the niche has also failed in *hard*.

More remarkable is that the intraspecific CSC coefficient has changed its sign, an indication that CSCs record a stressed environment that they may perceive as due to the presence of damaged tissue. This generates a phenotype different from that in *soft* [18], which accelerates cell division. On the other hand, the large and positive DCC intraspecific coefficient, *α*_*DD*_=1.83, implies a huge increase of the inhibitory signaling between DCCs with respect to *soft*. In this case, the discussion following Eq. 4 suggests for this system a maximum intrinsic DCC number smaller than unity, *D*_*c*_=1/*α*_*DD*_≃0.5 cells, meaning that on this substrate the DCC subpopulation would not be able to survive without the CSCs.

The general picture is that of a growing tumorsphere whose response to the substrate is to increase its cell number as fast as possible, aiming to reach a DCC fraction that equilibrates the niche, a goal that cannot be attained due to the presence of differentiation-inhibiting agents. The influence of the niche, as in [18], is thus a cornerstone for the biological interpretation of the model results.

### Control substrate

As mentioned in the previous section, we cannot expect the model to give a completely accurate description of the *control* substrate experiment, but its interpretation may shed light on the system dynamics. In this condition CSCs are allowed to freely differentiate. These cells record an environment where the proportion of DCCs increases monotonically and the population fractions should tend to those corresponding to niche equilibrium. However, Figs. 5 and 6 suggest that there is no limit to the increase of the DCC fraction. We conjecture that this behavior may be explained by migration: after the spheroid reaches a given size, cells start to migrate and the average number of DCCs recorded by each CSC does not increase. One consequence is that the CSC number remains stationary as shown in Fig. 5. In fact, their effective PDT is of about six months, i.e., they are generally quiescent, as they should.

Furthermore, note that the whole PDT leads to a duplication of the first 5 cells after 41 days (1/0.024), which is three times slower than the *soft* rate (15 days). To explain the rapid spheroid growth we need to consider the contribution of the interactions. From Table 1 we see that interactions favor DCCs and restrict CSCs proliferation. A more detailed analysis of the evolution of the two subpopulations reveals the following:

- CSCs: The positivity and very low absolute values of *α*_*SS*_ and *α*_*SD*_ ensure the stability of the CSC number. The dominant contribution to the change in the CSC number is given by the linear term, which yields |*p*_*s*_*r*|^−1^=185 days, meaning that the CSCs are quiescent during the whole experiment.
- DCCs: Approximating Eq. (1b) with *S*→0, we get D = 1/ *α*_*DD*_. Because *α*_*DD*_<0, the quadratic term always promotes DCC number growth. As mentioned in the case of *hard*, a negative sign in the intraspecific interaction parameter is related to signaling loss. Sphere disaggregation in *control* suggests that the *hard* substrate promotes migration, weakening cell-to-cell interactions. Given that the CSC pool remains constant while many cells move away from the spheroid, we can also conclude that the migrating cells are likely to be differentiated.

Of note, our analysis of these experiments implies that the absence of the stem cell growth factors in *control* leads to the disappearance of the feedback loop that plays such a crucial role in both *soft* and *hard*. The existence of the feedback loops detected in *soft* and *hard* can similarly be inferred from experiments carried out with the cancer lines SUM159, MCF-7, and T47D, which were also cultured with stem cell growth factors [9, 18].

Even if the CSC fractions are not far from unity in both *soft* and *hard*, the detailed reasons for their behavior are different. In both cases the cell subpopulations assist each other, generating a positive-feedback cycle that leads to continuous growth, an indication that cell-to-cell signaling is crucial to determine the process. The effect of the substrate on intraspecific interactions in *hard* is strong. There, CSCs are weakly promoting, but DCCs are so strongly inhibitory that the DCC population would disappear if it were not for the significant cooperation from the CSCs, which is expressed mainly through a considerable fraction of asymmetric divisions. The inhibition between DCCs is also likely to induce the phenotype change indicated by the large and negative value of *α*_*SD*_. The parameter *α*_*DS*_, which controls the influence of cancer stem cells on differentiated cancer cells, is always negative, and very strong so in *control*, suggesting that CSCs have a promoting and protective influence on DCCs, a phenomenon that was already observed by Kim and coworkers. These authors found that CSCs protect DCCs from anoikis promoting tumor formation when the two subpopulations are mixed [49]. The smaller magnitude of *α*_*DS*_ in the *soft* and *hard* experiments suggests that stem cell maintenance factors weaken, but do not cancel, this protective effect.

## Conclusion

The analysis of experimental data with our model confirms Wang’s conclusions and indicates that the substrate regulates the details of tumorsphere evolution and that a powerful engine of tumorsphere growth is the stem cell “memory" of its niche. By comparing growth on the *hard* and *soft* substrates, our analysis also confirms the observations that substrate stiffness promotes cancer cell proliferation (as recently reviewed by Nia et al. [50]. What is more interesting is that the evident differences between the parameters describing growth on the *hard* and *control* substrates indicate that the response of stem and non-stem cancer cells to an increase in substrate stiffness is likely to be mediated by different processes.

In summary, the ability of stem cells to sense their environment plays a crucial role in tumorsphere evolution. Our model has proven to be particularly useful at determining why substratum stiffness has a profound influence on the behavior of cancer stem cells, soft substrates favoring symmetric divisions and hard substrates leading to a large proportion of asymmetric doublings. In vivo studies are needed to further our understanding of niche processes under natural environments.

## Supplementary Information

**Additional file 1** Some consequences of equation (1). The onset of growth and the fate of a tumorsphere.

**Additional file 2** Distribution graphs from fitting procedure.

## Data Availability

The datasets used during the current study are available from the corresponding author on reasonable request.

## Abbreviations

CSC: Cancer stem cells
DCC: Differentiated cancer cells
RGS: Random grid search

## References

[1] Lapidot T (1994). A cell initiating human acute myeloid leukaemia after transplantation into SCID mice. Nature.

[2] Al-Hajj M, Clarke MF (2004). Self-renewal and solid tumor stem cells. Oncogene.

[3] Singh SK, Hawkins C, Clarke ID, Squire JA, Bayani J, Hide T (2004). Identification of human brain tumour initiating cells. Nature.

[4] Li C, Heidt DG, Dalerba P, Burant CF, Zhang L, Adsay V (2007). Identification of pancreatic cancer stem cells. Cancer Res.

[5] O’Brien CA, Pollett A, Gallinger S, Dick JE (2007). A human colon cancer cell capable of initiating tumour growth in immunodeficient mice. Nature.

[6] Eramo A, Lotti F, Sette G, Pilozzi E, Biffoni M, Di Virgilio A (2008). Identification and expansion of the tumorigenic lung cancer stem cell population. Cell Death Differ.

[7] Batlle E, Clevers H (2017). Cancer stem cells revisited. Nat Med.

[8] Jagust P, de Luxán-delgado B, Parejo-Alonso B, Sancho P (2019). Metabolism-Based Therapeutic Strategies Targeting Cancer Stem Cells. Front Pharmacol.

[9] Chen YC, Ingram PN, Fouladdel S, Mcdermott SP, Azizi E, Wicha MS (2016). High-throughput single-cell derived sphere formation for cancer stem-like cell identification and analysis. Sci Rep.

[10] Visvader JE, Lindeman GJ (2012). Cancer stem cells: Current status and evolving complexities. Cell Stem Cell.

[11] Taniguchi S, Elhance A, Van Duzer A, Kumar S, Leitenberger JJ, Oshimori N (2020). Tumor-initiating cells establish an IL-33–TGF- *β* niche signaling loop to promote cancer progression. Science.

[12] Stiehl T, Marciniak-Czochra A (2012). Mathematical modeling of leukemogenesis and cancer stem cell dynamics. Math Model Nat Phenom.

[13] Stiehl T, Baran N, Ho AD, Marciniak-Czochra A (2015). Cell division patterns in acute myeloid leukemia stem-like cells determine clinical course: A model predict patient survival. Cancer Res.

[14] Yang J, Plikus MV, Komarova NL (2015). The Role of Symmetric Stem Cell Divisions in Tissue Homeostasis. PLoS Comput Biol.

[15] Bessonov N, Pinna G, Minarsky A, Harel-Bellan A, Morozova N (2019). Mathematical modeling reveals the factors involved in the phenomena of cancer stem cells stabilization. PLoS ONE.

[16] Park JS, Kim HN, Kim DH, Levchenko A, Suh KY (2012). Quantitative analysis of the combined effect of substrate rigidity and topographic guidance on cell morphology. IEEE Trans Nanobiosci.

[17] Kumar S, Weaver VM. (2009). Mechanics, malignancy, and metastasis: The force journey of a tumor cell. Cancer Metastasis Rev.

[18] Benítez L, Barberis L, Condat CA (2019). Modeling tumorspheres reveals cancer stem cell niche building and plasticity. Physica A.

[19] Taloni A, Ben Amar M, Zapperi S, La Porta CAM (2015). The role of pressure in cancer growth. Eur Phys J Plus.

[20] Northcott JM, Dean IS, Mouw JK, Weaver VM (2018). Feeling Stress : The Mechanics of Cancer Progression and Aggression. Front Cell Dev Biol.

[21] La Porta CAM, Zapperi S, Sethna JP (2012). Senescent Cells in Growing Tumors: Population Dynamics and Cancer Stem Cells. PLoS Comput Biol.

[22] Agur Z, Kogan Y, Levi L, Harrison H, Lamb R, Kirnasovsky OU (2010). Disruption of a Quorum Sensing mechanism triggers tumorigenesis: A simple discrete model corroborated by experiments in mammary cancer stem cells. Biol Direct.

[23] Helmlinger G, Netti PA, Lichtenbeld HC, Melder RJ, Jain RK (1997). Solid stress inhibits the growth of multicellular tumor spheroids. Nat Biotechnol.

[24] Delsanto PP, Guiot C, Degiorgis PG, Condat CA, Mansury Y, Deisboeck TS (2004). Growth model for multicellular tumor spheroids. Appl Phys Lett.

[25] Koike C, McKee TD, Pluen A, Ramanujan S, Burton K, Munn LL (2002). Solid stress facilitates spheroid formation: Potential involvement of hyaluronan. Br J Cancer.

[26] Cheng G, Tse J, Jain RK, Munn LL (2009). Micro-environmental mechanical stress controls tumor spheroid size and morphology by suppressing proliferation and inducing apoptosis in cancer cells. PLoS ONE.

[27] Montel F, Delarue M, Elgeti J, Vignjevic D, Cappello G, Prost J (2012). Isotropic stress reduces cell proliferation in tumor spheroids. New J Phys.

[28] Delarue M, Montel F, Vignjevic D, Prost J, Joanny JF, Cappello G (2014). Compressive stress inhibits proliferation in tumor spheroids through a volume limitation. Biophys J.

[29] Desmaison A, Guill L, Triclin S, Wei P, Ducommun B, Lobjois V (2018). Impact of physical confinement on nuclei geometry and cell division dynamics in 3D spheroids. Sci Rep.

[30] Desmaison A, Frongia C, Grenier K (2013). Ducommun B„ Lobjois V. Mechanical stress impairs mitosis progression in multi-cellular tumor spheroids. PLoS One.

[31] Mills KL, Kemkemer R, Rudraraju S, Garikipati K (2014). Elastic Free Energy Drives the Shape of Prevascular Solid Tumors. PLoS ONE.

[32] Saha K, Keung AJ, Irwin EF, Li Y, Little L, Schaffer DV (2008). Substrate modulus directs neural stem cell behavior. Biophys J.

[33] Chen A, Wang L, Liu S, Wang Y, Liu Y, Wang M (2018). Attraction and Compaction of Migratory Breast Cancer Cells by Bone Matrix Proteins through Tumor-Osteocyte Interactions. Sci Rep.

[34] Sun M, Chi G, Xu J, Tan Y, Xu J, Lv S (2018). Extracellular matrix stiffness controls osteogenic differentiation of mesenchymal stem cells mediated by integrin *α*5. Stem Cell Res Ther.

[35] Nakano T, Okaie Y, Kinugasa Y, Koujin T, Suda T, Hiraoka Y (2020). Roles of Remote and Contact Forces in Epithelial Cell Structure Formation. Biophys J.

[36] Discher DE, Janmey P, Wang YL (2005). Tissue cells feel and respond to the stiffness of their substrate. Science.

[37] Engler AJ, Sen S, Sweeney HL, Discher DE (2006). Matrix Elasticity Directs Stem Cell Lineage Specification. Cell.

[38] Choi HY, Yang GM, Dayem AA, Saha SK, Kim K, Yoo Y (2019). Hydrodynamic shear stress promotes epithelial-mesenchymal transition by downregulating ERK and GSK3 *β* activities. Breast Cancer Res.

[39] Smith Q, Chan XY, Carmo AM, Trempel M, Saunders M, Gerecht S (2017). Compliant substratum guides endothelial commitment from human pluripotent stem cells. Sci Adv.

[40] Weiswald LB, Bellet D, Dangles-Marie V (2015). Spherical cancer models in tumor biology. Neoplasia (New York, NY).

[41] Wang J, Liu X, Jiang Z, Li L, Cui Z, Gao Y (2016). A novel method to limit breast cancer stem cells in states of quiescence, proliferation or differentiation: Use of gel stress in combination with stem cell growth factors. Oncol Lett.

[42] Britton NF (2003). Essential Mathematical Biology.

[43] Rodriguez-Brenes IA, Komarova NL, Wodarz D (2011). Evolutionary dynamics of feedback escape and the development of stem-cell-driven cancers. Proc Natl Acad Sci.

[44] Hamidi H, Ivaska J (2018). Every step of the way: integrins in cancer progression and metastasis.. Nat Rev Cancer.

[45] Vellon L, Menendez JA, Lupu R (2005). *α**v**β*3 integrin regulates heregulin (HRG)-induced cell proliferation and survival in breast cancer. Oncogene.

[46] Vellon L, Menendez JA, Lupu R (2006). A bidirectional “ *α**v**β*3 integrin-ERK1/ERK2 MAPK” connection regulates the proliferation of breast cancer cells. Mol Carcinog.

[47] Pollard SM, Yoshikawa K, Clarke ID, Danovi D, Stricker S, Russell R (2009). Glioma Stem Cell Lines Expanded in Adherent Culture Have Tumor-Specific Phenotypes and Are Suitable for Chemical and Genetic Screens. Cell Stem Cell.

[48] Videla Richardson GA, Garcia CP, Roisman A, Slavutsky I, Fernandez Espinosa DD, Romorini L (2016). Specific Preferences in Lineage Choice and Phenotypic Plasticity of Glioma Stem Cells under BMP4 and Noggin Influence. Brain Pathol.

[49] Kim SY, Hong SH, Basse PH, Wu C, Bartlett DL, Kwon YT (2016). Cancer Stem Cells Protect Non-Stem Cells From Anoikis: Bystander Effects. J Cell Biochem.

[50] Nia HT, Munn LL, Jain RK (2020). Physical traits of cancer. Science.

