# Supplementary material for: Understanding the influence of substrate when growing tumorspheres
Source: BMC Cancer. 2021 Mar 15;21:276. doi: 10.1186/s12885-021-07918-1 (PMC7962376; doi:10.1186/s12885-021-07918-1)
Supplement: Supplementary file 1 — Additional file 1 Some consequences of equation (1). The onset of growth and the fate of a tumorsphere. [file 12885_2021_7918_MOESM1_ESM.pdf]

## Additional file 1:
## Some consequences of Equation (1).
## The onset of growth and the fate of a tumorsphere

The system of equations (1) can be solved numerically, yielding solutions that describe the time evolution of the subpopulations. The equilibrium values of the subpopulations are obtained by setting the time derivatives in Eqs. (1) equal to zero. We will refer to these solutions as "equilibrium points". Our mathematical model has three relevant equilibrium points [18] First, a zero population point (0,0) corresponding to the complete absence of cells. Second, a differentiated cell point $(0, 1/\alpha_{DD})$, corresponding to a system that contains $1/\alpha_{DD}$ DCCs and no CSCs. Finally, a coexistence point $(S^*, D^*)$, which corresponds to a mixture of the two kind of cells, whose numbers are:

$$S^* = \alpha_{DD}(p_s - p_d) - \alpha_{SD}p_s(\alpha_{SS}\alpha_{DD} - \alpha_{SD}\alpha_{DS})p_s, \tag{A1a}$$

$$D^* = \alpha_{SS}p_s - \alpha_{DS}(p_s - p_d)(\alpha_{SS}\alpha_{DD} - \alpha_{SD}\alpha_{DS})p_s. \tag{A1b}$$

As shown in [18], this system undergoes a transcritical bifurcation. This means that there is a parameter range where a small change in one parameter leads to a switch in the tumor fate from the state containing only differentiated cells to the state of coexistence. The end point of the tumorsphere evolution is the differentiated cell equilibrium if

$$\frac{\alpha_{SD}}{\alpha_{DD}} > \left(1 - \frac{p_d}{p_s}\right). \tag{A2}$$

The tumor will reach the coexistence point if this inequality is reversed. These solutions have two interesting features: The DCC point occurs only if $\alpha_{DD} > 0$, when the differentiated cancer cells inhibit each other. Strengthening the inhibition, the fixed point moves towards the origin implying a smaller final size. The location of this point, but not its stability, is independent of the other parameters. For small values of $p_s$, when few stem cells divide symmetrically, and $\alpha_{SD} > 0$, the DCC point is stable, while the unstable coexistence point moves to very large values, as $1/p_s$, c.f. Eqs. (A1). The initial evolution of a small system can be approximately described using a linearized version of our dynamic equations. Assuming $S(0) = S_0$ and $D(0) = D_0$ and linearizing either Eqs. (1) or their non-dimensional counterparts, we can find the initial shape of the trajectories in the $S{-}D$ plane. In the special case $S_0 = 0$, $D(t) = D_0 \exp(rt)$, and the tumor begins to grow exponentially along the DDC line. In the more useful (experimental) situation $S_0 \neq 0$, and,

$$S(t) = S_0 \exp[-(p_d - p_s)rt]. \tag{A3}$$

The trajectory in the $S - D$ plane starts as,

$$D(t) = \frac{(D0 + S0)}{S0} S(t)^{1/(p_s - p_d)} - S(t). \tag{A4}$$
