# Supplementary material for: Understanding the influence of substrate when growing tumorspheres
Source: BMC Cancer. 2021 Mar 15;21:276. doi: 10.1186/s12885-021-07918-1 (PMC7962376; doi:10.1186/s12885-021-07918-1)
Supplement: Supplementary file 2 — Additional file 2 Distribution graphs from fitting procedure. [file 12885_2021_7918_MOESM2_ESM.pdf]

Additional file 2:
Distribution graphs from fitting procedure.

In Figs. A, B and C, we illustrate the result of implementing our fitting method as box-plots of the distributions of the obtained data. For each parameter, specified in the upper-right corner of the box-plot, we mark the values obtained and draw the box where half the points fall and the median of the distribution. The latter becomes the chosen value of the corresponding parameter.

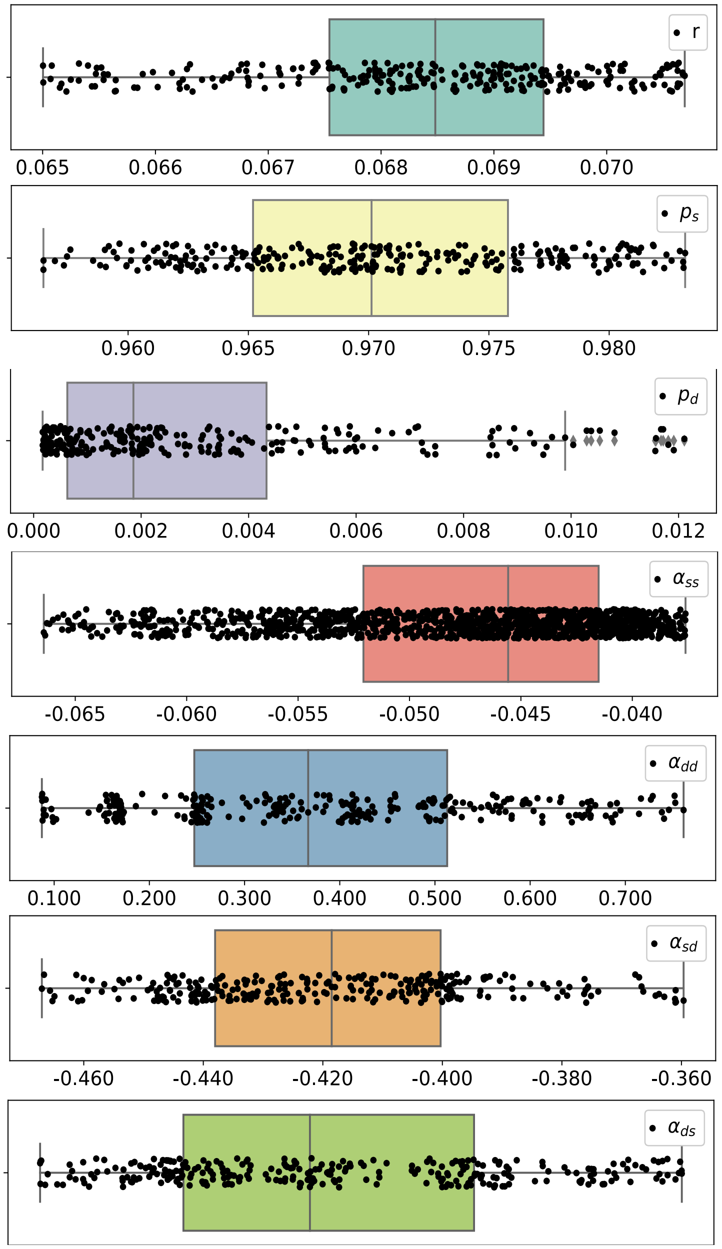

Figure A: Parameter distributions of *soft* experiment. Boxplot representation of the fitting parameter distributions obtained by RGS for the *soft* experiment. The box in each panel contains 50% of the data around the center of the corresponding distribution. The middle vertical line indicates the median of the distribution.

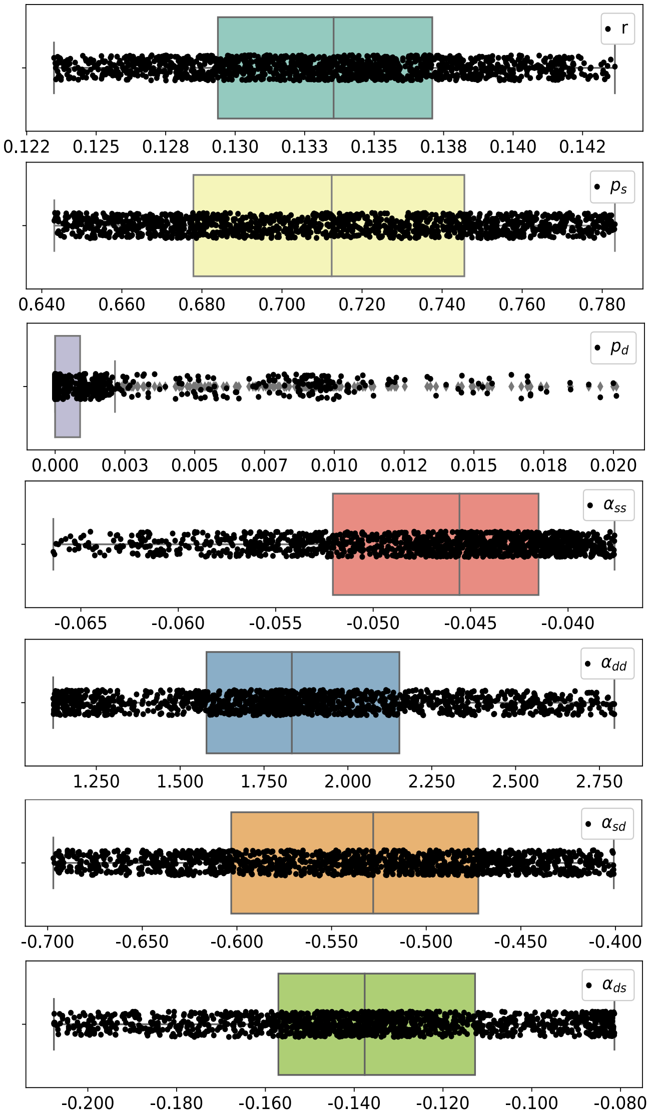

Figure B: Parameter distributions of *hard* experiment. Boxplot representation of the fitting parameter distributions obtained by RGS for the *hard* experiment. The box in each panel contains 50% of the data around the center of the corresponding distribution. The middle vertical line indicates the median of the distribution.

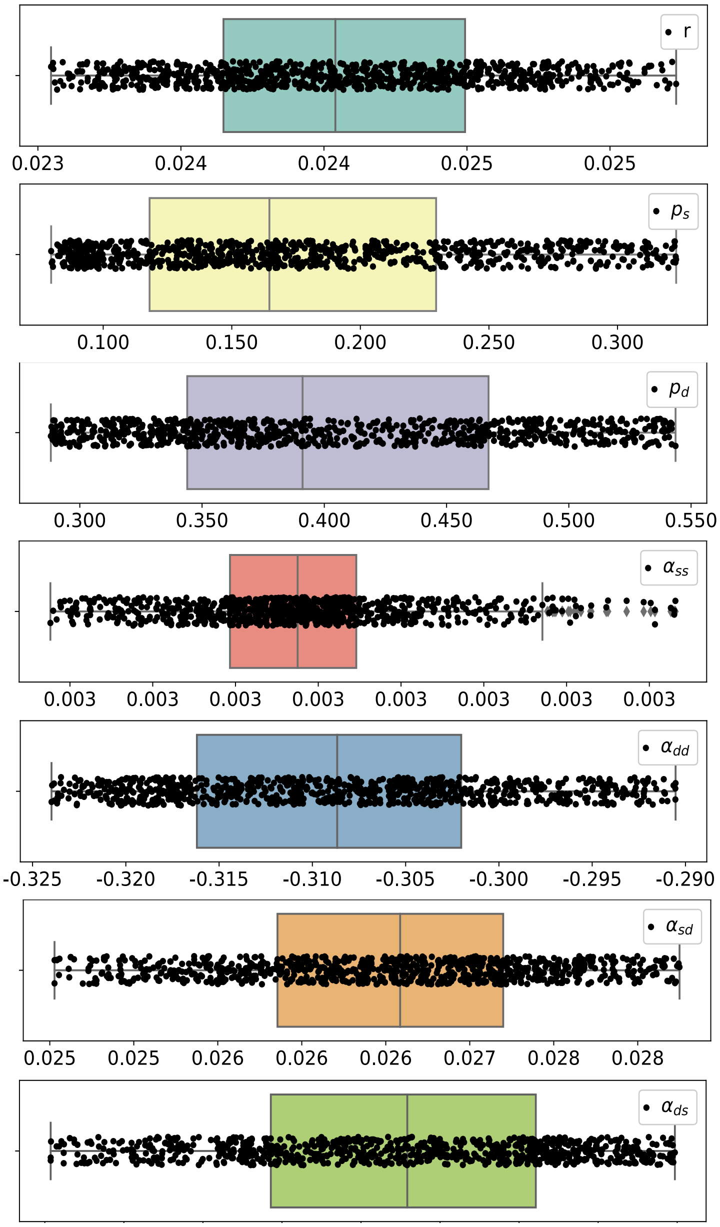

Figure C: Parameters distributions of *control* experiment. Boxplot representation of the fitting parameter distributions obtained by RGS for the *control* experiment. The box in each panel contains 50% of the data around the center of the corresponding distribution. The middle vertical line indicates the median of the distribution.
